# Supplementary material for: TDP-43 Regulates Rab4 Levels to Support Synaptic Vesicle Recycling and Neuromuscular Connectivity in Drosophila and Human ALS Models
Source: Int J Mol Sci. 2025 Nov 14;26(22):11030. doi: 10.3390/ijms262211030 (PMC12652574; doi:10.3390/ijms262211030)
Supplement: Supplementary file 1 [file ijms-26-11030-s001.zip › ijms-3922116-supplementary.pdf]

Supplemental Material

TDP-43 regulates Rab4 levels to support synaptic vesicle recycling and neuromuscular connectivity in Drosophila and human ALS models

Monsurat Gbadamosi[1], Giulia Romano[1], Michela Simbula[2], Giulia Canarutto[1,3], Linda Ottoboni[4,5], Stefania Corti[4,6] and Fabian Feiguin[7]*

[1] International Centre for Genetic Engineering and Biotechnology, Padriciano 99, 34149 Trieste, Italy.

[2] Institute for Genetic and Biomedical Research, The National Research Council (CNR); 09042 Monserrato, Italy.

[3]Department of Life Sciences, University of Trieste, 34127, Trieste, Italy.

[4] Dino Ferrari Center, Department of Pathophysiology and Transplantation, University of Milan, Italy.

[5]Neurology Unit, Fondazione IRCCS Ca' Granda Ospedale Maggiore Policlinico, Milan, Italy.

[6] Neuromuscular and Rare Diseases Unit, Fondazione IRCCS Ca' Granda Ospedale Maggiore Policlinico, Milan, Italy.

[7]Department of Life and Environmental Sciences, University of Cagliari, 09042 Monserrato, Italy.

* Corresponding author.
Email: fabian.feiguin@unica.it

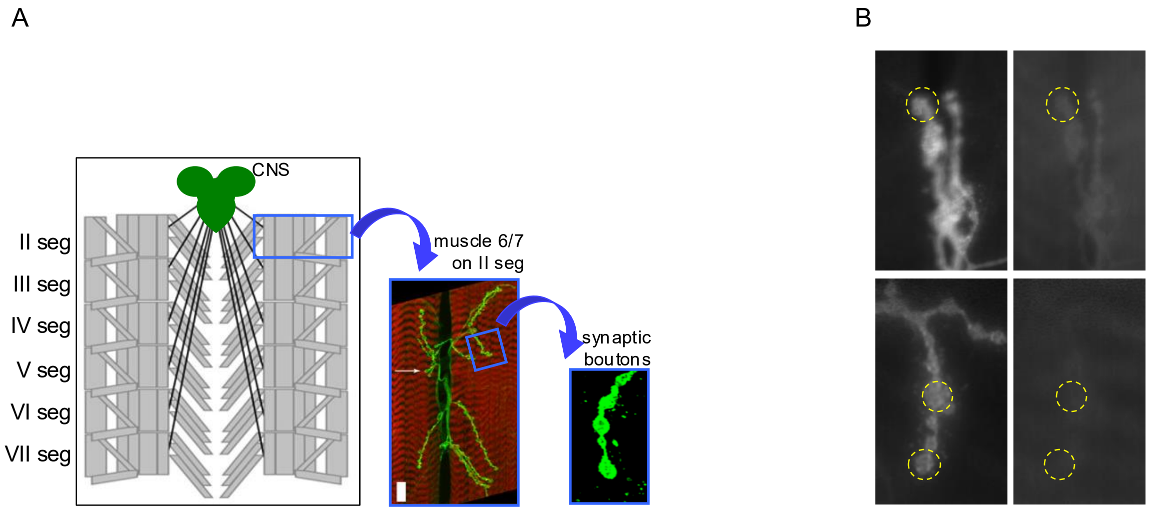

Supplemental Figure S1. Synaptic vesicle loading and unloading analysis at neuromuscular junctions of third-instar larvae using FM1-43 dye.

(**A**) Schematic representation of the anatomical organization of a third instar larva following dissection, illustrating the repeated segmental pattern observed throughout the body, in which muscle architecture is conserved. To ensure reproducibility and minimize variability related to anatomical differences, analyses were restricted to muscles 6 and 7 in the second abdominal segment. In the second panel, these muscles are shown in red, and the analyzed synaptic terminal is highlighted in green. A third panel provides a magnified view of the synaptic bouton structures. This is a representative cartoon, created with unrelated images for the sole purpose of explaining the anatomy."(**B**) A region of interest (ROI) was defined around each synaptic bouton for quantitative analysis. Signal intensity was recorded during the loading phase, and the same ROI was subsequently applied to the image acquired after terminal unloading. Throughout the experiment, the tissue remained fixed on the microscope stage and the optical configuration was kept constant to ensure that the same anatomical region was maintained across all recordings. The mean loading signal of the control was set to 100%, and all other values were expressed as percentages normalized to the wild-type mean. For unloading measurements, the proportion of signal loss relative to the initial loading signal was calculated and subtracted from the initial value. Quantification of the space occupied by sub-boutonic structures was performed on the loading images. A ROI was drawn around each bouton, and the proportion of signal exceeding the background threshold was computed. For each synaptic bouton, the percentage of area containing signal above the background level (positive) and that equal to or below background (negative) was determined. Individual bouton measurements were then averaged to generate the mean values presented in the graph.

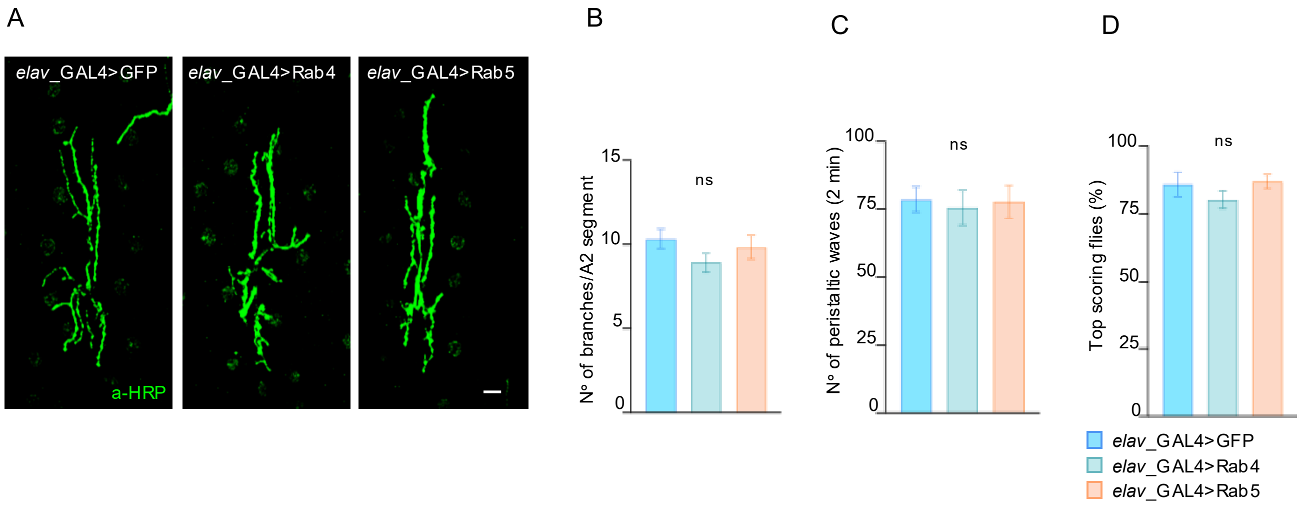

Supplemental Figure S2. Rab4 overexpression does not affect NMJ morphology or locomotor behaviours in wild-type flies.
(**A**) Confocal image of third instar NMJ terminals of muscle 6/7 second segment in *elav*_GAL4>GFP , *elav*_GAL4>Rab4 and *elav*_GAL4>Rab5 stained with anti-HRP (in green); *n*>12 larvae. Relative quantification of branches number in (**B**). (**C**) Number of peristaltic larval waves of third instar larvae during 2 minutes of *elav*_GAL4>GFP , *elav*_GAL4>Rab4 and *elav*_GAL4>Rab5. *n*>15 larvae (**D**) A climbing assay was performed on 7-day-old adult flies of *elav*_GAL4>GFP , *elav*_GAL4>Rab4 and *elav*_GAL4>Rab5 genotypes to assess locomotor performance. *n*>60 flies. ns=not significant, calculated by one-way ANOVA, error bars SEM. Scale bar 40µm.
